# Health Education Workforce: Opportunities and Challenges

**DOI:** 10.5888/pcd15.180045

**Published:** 2018-06-28

**Authors:** Rebecca A. Bruening, Fátima Coronado, M. Elaine Auld, Gabrielle Benenson, Patricia M. Simone

**Affiliations:** 1 Association of Schools & Programs of Public Health Fellow, Division of Scientific Education and Professional Development, Centers for Disease Control and Prevention, Atlanta, Georgia; 2 Division of Scientific Education and Professional Development, Centers for Disease Control and Prevention, Atlanta, Georgia; 3 Society for Public Health Education, Washington, District of Columbia

Public health is facing unprecedented opportunities and challenges. Health departments face shifts from less clinical service delivery to increased population-based services to address the growing burden of chronic diseases (eg, obesity prevention, tobacco and drug use prevention) and new responsibilities to collaborate with other sectors in conducting community needs assessments and data sharing (1–3). State and local health departments continue to be challenged by health policy changes, reduced budgets, and difficulty recruiting and retaining staff (2). These challenges need to be met with a public health workforce of adequate size, composition, distribution, and skills. Formally trained health educators are an important but often underutilized part of the workforce needed to meet such challenges. Although various health workers inform the public, many employers are unaware of the professional training and roles of health educators (4). Health educators (also referred to as health education specialists) address chronic and other conditions by applying their competencies to the design and execution of behavioral health and policy or systems interventions (4). This essay highlights how the skill sets of health educators can address current and future public health challenges, the need for improved health educator workforce data, and a call to action for various stakeholders to optimally deploy health educators to improve the public’s health.

## Skill Sets of Health Educators to Meet Public Health Needs

Health educators are uniquely positioned to address public health needs by deploying their training and competencies in the application of behavioral theories across a wide range of interventions designed to improve population health. Health educators use a holistic approach to changing health behaviors, implementing evidence-based interventions and adapting to changing population needs. For example, health educators can help multicultural populations with access to and use of the health system by improving their health insurance literacy and understanding of enrollment options and can conduct community health needs assessments required for nonprofit hospitals, modify policies or systems to improve access to preventive health services, and strengthen clinical and community linkages (5). In addition, health educators can develop and implement training programs for professionals and consumers, market health programs and services, conduct evaluation research, analyze data and educate populations about wellness behaviors (eg, vaccination campaigns, opioid abuse prevention programs), provide consultation to health agencies about different topics (eg, smoking prevention and cessation efforts), and apply their skills in cross-cultural communication and community organizing (eg, prenatal programs for expectant mothers in diverse communities) (5).

A health educator has training in public health, health promotion, or community health at the bachelor’s, master’s, or doctoral levels. To maintain the highest level of competence in the health education workforce, health educators can obtain additional certifications such as the Certified Health Education Specialist (CHES) and Master Certified Health Education Specialist (MCHES), both awarded through the National Commission for Health Education Credentialing to people who have met academic preparation and examination qualifications, which requires that the person remains up to date with education requisites, acquiring at least 75 continuing education contact hours every 5 years.

## Health Educator Employment Data

Although many people are professionally prepared as health educators, available data are limited about where they work, how they contribute, or how they can be better deployed to serve public health needs. Despite the US Department of Labor’s Standard Occupational Classification of health educators, the definition is not widely embraced throughout the government public health system. Because job descriptions often include functions of other health workers, including health communicators and community health workers, some employers might have difficulty distinguishing health educators from other professionals. Furthermore, professional identification and occupational classifications can differ substantially among health educators. Employers often use other titles to classify positions for health educators, likely underestimating the number of health educators working under alternative job titles and complicating efforts to collect and interpret workforce data (4).

Describing the health educator workforce is further challenged by limited employment data available across all industries, a concern common to many public health professions (6). Health educators work in diverse settings, including governmental public health agencies, health care organizations, schools and colleges, community organizations, and corporations. In 2015, the US Bureau of Labor Statistics reported that 57,750 people were employed as health educators, compared with 63,320 in 2006; the industries with the highest levels of health educator employment were government (22%), hospitals (21%), and ambulatory health care services (16%) (7). However, it is possible that this trend could be because these industries might have a similar approach to job classifications, as noted earlier.

In a 2016 survey of local health departments, 53% of respondents indicated that health education is a high-priority occupation in their agencies, and 76% reported the need for more health educator positions (8). Although the US Department of Labor predicted growth in the number of health educators attributable to increased focus on prevention driven by changes in health care systems and insurance coverage (7), from 2010 to 2013 the numbers remained the same at the local government level and dropped in state health departments (9). Part of the reason might be ambiguity regarding the competencies of health educators and community health workers. The 2 occupations have distinct standard occupational classifications, but their descriptions are combined in the US Department of Labor Occupational Handbook. Although the two have complementary roles in addressing community and individual health needs, their training and skill levels differ.

## Call to Action

Health educators play a vital role in addressing public health concerns, but opportunities exist for even greater contributions. As the nation develops Healthy People 2030 objectives, improvements are needed in educating employers about health educator competencies, collecting and analyzing health educator workforce data, and strengthening health educator professional preparation and in-service training. We propose a set of actions for the health education community and other stakeholders in schools and programs of public health, government public health agencies, health systems, and professional associations, to help address these challenges.

**Skills**. The health educator profession should continue to incorporate broad-based skills that match current public health needs into professional preparation and continuing education. To keep pace with emerging public health challenges, government public health workers, including health educators, increasingly require more strategic skills that address the social, community-based, and economic determinants of health (10). Training needs include understanding systems thinking and identifying high-impact interventions, changing management approaches to scale programs in response to evolving environments, using data for decision making, identifying and solving problems and evaluating results, engaging underrepresented populations, acquiring human and fiscal resource management knowledge, and addressing public health concerns while engaging a broader audience of policy and decision makers (10).

**Advocacy**. The health educator community can be effective advocates for their own profession, educating their human resource departments about expected competencies of professionally trained health educators, the differences between health educators and community health workers, certifications, recruitment avenues, and the contributions of health educators to the bottom line (5). They can help others in the public health community recognize the value of health education by promoting certification as a quality assurance mechanism for the field, disseminating results about health educators’ contributions to health outcomes, and regularly assessing competencies needed for health educators to meet contemporary public health challenges. Similarly, they can help inform grant funders as well as public and private employers on how the use of contemporary health educator skills can help them achieve their population health goals.

**Data**. Data from health educator-related academic programs regarding the employment outcomes of their graduates can help assess the quality of professional preparation in terms of employment job duties. To complement data from academic programs, public and private workforce surveys should include questions about professional training and link to occupational classifications by using an established taxonomy (6). Such data are crucial to determine whether the supply of professionally prepared health educators is sufficient to meet demand and to guide decision makers and researchers to identify workforce gaps, improve workforce development, and recruit and retain health educators in sectors where they are most needed. Because the challenges of describing the health education workforce are similar to those of the larger public health workforce, health education stakeholders should engage with other public health partners on innovative, cross-cutting solutions for shared workforce priorities, addressing them in a coordinated approach.

With shrinking resources and mounting public health demands, public health agencies and organizations must collaborate to deploy the capabilities of all public health workers, including health educators, at their highest levels of competence. Trained health educators are vital to addressing public health needs and contributing to healthy communities. By incorporating broad-based competencies into professional preparation, promoting health educator skills and competencies to employers, and improving enumeration and tracking of health educators and other public health professions in the public health workforce, we will be poised to achieve Healthy People 2030 objectives, for the nation and beyond.
